# Synthesis of New Functionalized Indoles Based on Ethyl Indol-2-carboxylate

**DOI:** 10.3390/molecules21030333

**Published:** 2016-03-10

**Authors:** Ahmed T. A. Boraei, El Sayed H. El Ashry, Assem Barakat, Hazem A. Ghabbour

**Affiliations:** 1 Chemistry Department, Faculty of Science, Suez Canal University, Ismailia 41522, Egypt; 2 Chemistry Department, College of Science, King Saud University, P. O. BOX 2455, Riyadh 11451, Saudi Arabia; ambarakat@ksu.edu.sa; 3 Chemistry Department, Faculty of Science, Alexandria University, P. O. Box 426, Ibrahimia, Alexandria 21321, Egypt; eelashry60@hotmail.com; 4 Department of Pharmaceutical Chemistry, College of Pharmacy, King Saud University, P. O. Box 2457, Riyadh 11451, Saudi Arabia; ghabbourh@yahoo.com; 5 Department of Medicinal Chemistry, Faculty of Pharmacy, Mansoura University, Mansoura 35516, Egypt

**Keywords:** ethyl indol-2-carboxylate, alkylation, hydrazinolysis, single-crystal X-ray diffraction

## Abstract

Successful alkylations of the nitrogen of ethyl indol-2-carboxylate were carried out using aq. KOH in acetone. The respective *N*-alkylated acids could be obtained without separating the *N*-alkylated esters by increasing the amount of KOH and water. The use of NaOMe in methanol led to transesterification instead of the alkylation, while the use of NaOEt led to low yields of the *N*-alkylated acids. Hydrazinolysis of the ester gave indol-2-carbohydrazide which then was allowed to react with different aromatic aldehydes and ketones in ethanol catalyzed by acetic acid. Indol-2-thiosemicarbazide was used in a heterocyclization reaction to form thiazoles. The new structures were confirmed using NMR, mass spectrometry and X-ray single crystal analysis.

## 1. Introduction

Indole derivatives have been a topic of substantial research interest and continue to be one of the most active areas of heterocyclic chemistry, particularly due to their natural occurrence and pharmacological activities [1]. Indole derivatives also occur widely in many natural products such as those obtained from plants [2], fungi [3], and marine organisms [4]. The isolation, biological evaluation, and chemical properties of natural products have attracted the attention of organic chemists, medicinal chemists, biologists and pharmacists as well as led to optimization of highly efficient and economical synthetic routes.

**Figure 1:**
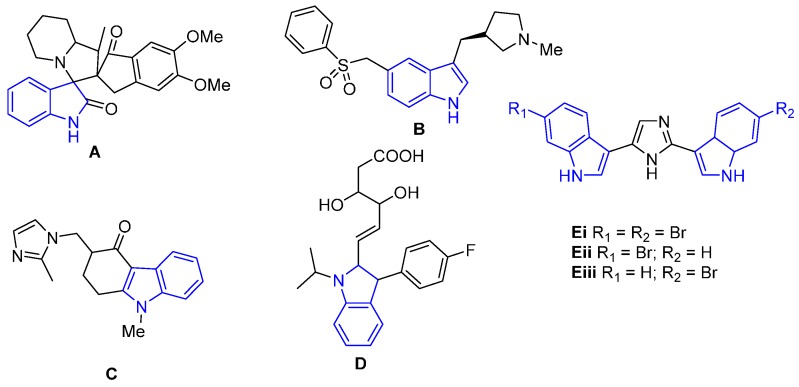
Biologically active compounds incorporating an indole scaffold.

At present there are several thousand indole alkaloids described, including simple and more complex functionalized indole compounds [5]. The growing importance of substituted indoles (Figure 1) in the field of medicinal chemistry as potential chemotherapeutic agents and their implication for pro-drug design have been previously reported [6,7,8,9,10,11,12,13,14,15].

The indazoline **A** is an indole derivative inhibitor of acetylcholinesterase used to treat Alzheimer’s disease [16]. The indole derivative eletriptan (**B**) is an anti-migraine drug. A process route for the synthesis of eletriptan published by Pfizer starts from a preformed bromoindole [17]. Fluvastatin (**C**) is a member of the statin drug class, used to treat hypercholesterolemia and to prevent cardiovascular diseases. It has also been shown to exhibit antiviral activity against hepatitis C [18]. Ondansetron (**D**) is a indole derivative used mainly as an antiemetic [19]. It is indicated for the prevention of acute nausea and vomiting associated with cancer chemotherapy [20].

Bis-indole alkaloids are an important structural class due to their high degree of biological activity. For example, the nortopsentins **Ei-iii** exhibit *in vitro* cytotoxicity against P388 cells with IC_50_ (inhibitory concentration) values of 7.6, 7.8, and 1.7 µg/mL, respectively [21,22,23].

Given the significant pharmacological activities associated with these heterocycles, and in order to contribute to the development of the chemistry of indole [24,25,26,27,28,29,30,31,32], we were interested in the synthesis of new heterocyclic polyfunctional indole derivative systems using alkylation reactions.

## 2. Results

Alkylation of the nitrogen of the indole ring in indole-containing compounds requires strong bases to generate the indole anion [33]. Protecting the nitrogen of the indole ring in ethyl 1*H*-indol-2-carboxylate requires special care to avoid the ester hydrolysis before the alkylation. KOH in anhydrous DMSO was used for the alkylation of nitrogen of the indole esters [34]. Herein, we describe the alkylation of the indole nitrogen using aq. KOH in acetone. In this method we can control the reaction to give the alkylated esters or the alkylated acids in the same reaction process, with the additional benefit of the ease of solvent removal after reaction completion.

**Scheme 1:**
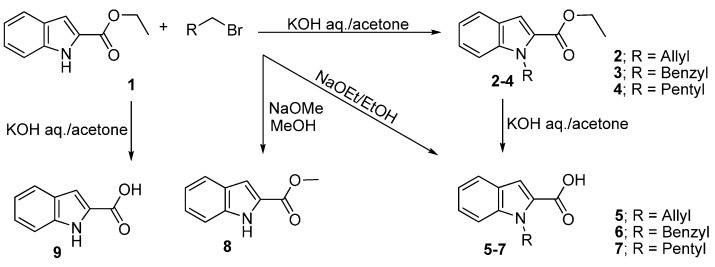
Alkylation of indole nitrogen, transesterification and ester hydrolysis.

Reaction of ethyl indol-2-carboxylate (**1**) with allyl bromide and benzyl bromide in the presence of aq. KOH (3.0 mmol/0.1 mL H_2_O/10 mL acetone) and stirring for two hours at 20 °C afforded ethyl 1-allyl-1*H*-indole-2-carboxylate (**2**) and ethyl 1-benzyl-1*H*-indole-2-carboxylate (**3**) in excellent yields. The corresponding alkylated carboxylic acids 1-allyl-1*H*-indol-2-carboxylic acid (**5**) and 1-benzyl-1*H*-indol-2-carboxylic acid (**6**) were obtained in high yields directly without separating the alkylated esters by increasing the amount of aq. KOH and refluxing for one hour. Alkylation with amyl bromide seems to be slow, since it took about eight hours to give ethyl 1-pentyl-1*H*-indole-2-carboxylate (**4**) under the same conditions. Moreover, a considerable amount of 1*H*-indol-2-carboxylic acid (**9**) was detected. The alkylated acids **5**–**7** were also obtained in excellent yields from the hydrolysis of the respective esters **2**–**4** using aqueous KOH in acetone**.** The use of NaOMe in methanol led to transesterification to afford methyl indol-2-carboxylate (**8**) instead of NH alkylation, whereas using NaOEt in ethanol gave the acids **5** and **6** in low to moderate yields in the case of the of **1** with allyl and benzyl bromides whereas, in case of amyl bromide a high yield of 1*H*-indol-carboxylic acid **9** was obtained (Scheme 1, Table 1).

**Scheme 2:**
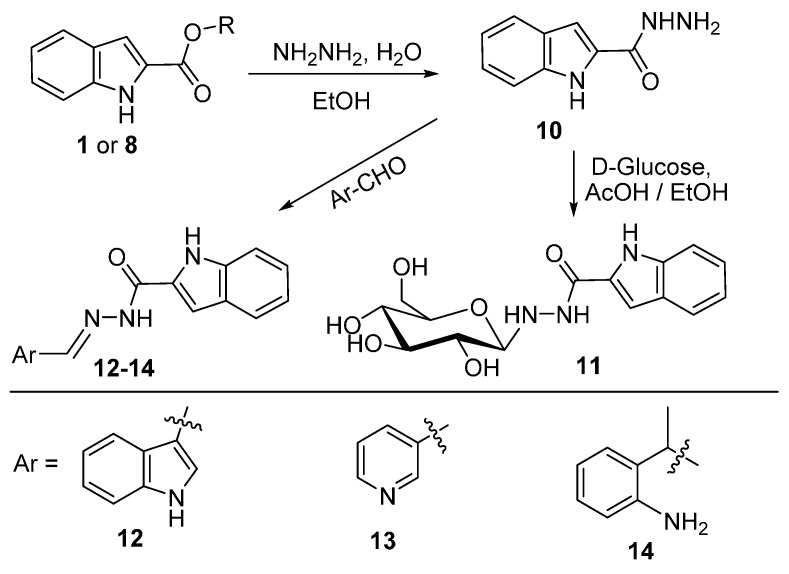
Reaction of indol-2-carbohydrazide **10** with aldehydes and ketones.

Hydrazinolysis of either ethyl or methyl esters **1** or **8** afforded indol-2-carbohydrazide (**10**). The hydrazide **10** was reacted with D-glucose, indol-3-carboxyaldehyde, pyridine-3-carboxyaldehyde and 2′-aminoacetophenone in ethanol and drops of acetic acid to yield *N*′-β-d-glucopyranosyl-1*H*-indole-2-carbohydrazide (**11**), *N*′-((1*H*-indol-3-yl)methylene)-1*H*-indole-2-carbohydrazide (**12**), *N*′-(pyridin-3-ylmethylene)-1*H*-indole-2-carbohydrazide (**13**) and *N*′-(1-(2-aminophenyl)ethylidene)-1*H*-indole-2-carbohydrazide (**14**), respectively (Scheme 2).

**Scheme 3:**
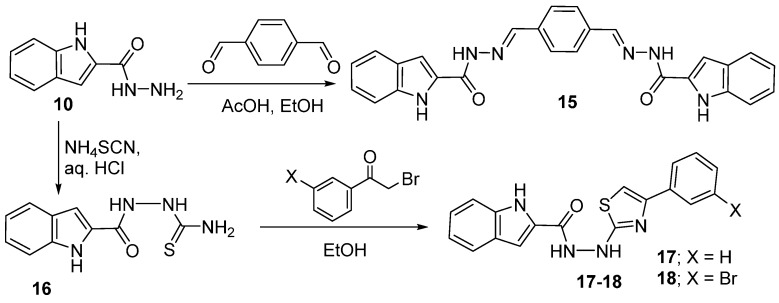
Synthesis of thiazoles **17** and **18**.

Moreover, hydrazide **10** was reacted with terephthalaldehyde under the same conditions to give the bis-indolyl product **15**. A thiosemicarbazide **16** was obtained from the hydrazide **10** and served as adduct for cyclization with two phenacyl bromides to afford indolylcarbonylhydrazino-thiazoles **17** and **18** (Scheme 3).

### 2.1. Structural Analysis

All NMR spectra showed the indole CH protons between δ 7.00 and 7.70 ppm and all indole carbons from δ 103.0 to 138.0 ppm.

### 2.2. Alkylated Ester Analysis

The formation of ethyl *N*-alkylated indol-2-carboxylates **2**–**4** was confirmed by the disappearance of the indole NH proton signal from the ^1^H-NMR of these compounds, the presence of ethoxy group signals (-OCH_2_CH_3_) at δ 1.30 and 4.30 ppm and the respective carbons in ^13^C-NMR at δ 15.0 and 46.7 ppm in addition to the ester carbonyl group around δ 162.0 ppm. Moreover, new signals appeared in the NMR spectra which are characteristic for the new groups and can be summarized as follows: in compound **2** the signals of the allyl group appeared as two doublets at δ 4.81 and 5.06 ppm for the olefinic CH_2_ with coupling constants δ 16.8 and 10.2 Hz, respectively, the NCH_2_ appeared at 5.23 ppm and the corresponding carbon (NCH_2_) appeared at δ 46.7 ppm, whereas, the remaining olefinic CH appeared as multiplet at δ 5.97–6.07 ppm. The benzylated ester **3** showed the NCH_2_ protons as a singlet at δ 5.87 ppm and the respective carbon at δ 47.6 ppm in addition to the phenyl protons and carbons. The pentylated ester **4** showed characteristic signals for the pentyl group protons at δ 0.83, 1.23–1.29, 1.68–1.70 and 4.55 ppm and the corresponding carbons appeared at δ 14.3, 22.3, 28.8, 30.4 and 44.4 ppm.

### 2.3. Hydrolyzed Ester Data Analysis

Hydrolysis of the esters with time was deduced from the disappearance of the signals of the ethoxy group in the NMR of **5**–**7** and instead a new broad signal appeared around δ 12.90 ppm for COOH, in addition to characteristic allyl, benzyl and pentyl signals.

Transesterification and formation of the methyl ester **8** was confirmed by ^1^H-NMR by observing a singlet signal at δ 3.88 ppm for the ester methyl group and the indole NH at δ 11.91 ppm. The respective methyl carbon appeared in ^13^C-NMR at δ 52.2 ppm and the ester carbonyl appeared at δ 162.3 ppm. The acid **9** lacked any alkyl signals and showed the C=O at δ 163.0 ppm, in addition to the remaining expected protons and carbons.

### 2.4. Hydrazinolysis of the Esters and the Related Products Analysis

Hydrazinolysis of either indol-2-carboxylate **1** or **2** led to the formation of indol-2-carboxylic acid hydrazide (**10**). The structure of **10** was confirmed by NMR which showed only two signals at δ 4.52 and 9.78 ppm for the -NH-NH_2_ group, whereas the carbonyl group appeared at δ 161.7 ppm. The ^1^H-NMR (DMSO-*d*_6_ + D_2_O) of **11** showed the anomeric proton as a doublet at 3.87 ppm with a coupling constant value of 8.7 Hz which confirms the β-configuration. The corresponding anomeric carbon appeared in ^13^C-NMR at 90.9 ppm and the carbonyl carbon signal appeared at 161.3 ppm.

The NMR spectra of **12**, **13** showed the -CH=N- proton around δ 8.60 ppm whereas, the NMR of **14** showed the methyl protons at 2.43 ppm and the respective methyl carbon at 15.5 ppm in addition to the remaining aromatic signals. The NMR spectra of **15** showed a singlet signal at δ 8.51 ppm for the two –CH=N- protons and two signals at δ 11.86 and 12.02 ppm for indole and hydrazide NHs, besides all aromatic protons and carbons signals.

### 2.5. Thiazole Structural Analysis

The ^1^H-NMR spectra of thiazoles **17** and **18** showed all aromatic CH protons of thiazole, indole and phenyl in the range of δ 7.07–7.83 ppm, while the three NH protons appeared as broad signals at δ 9.69, 10.87 and 11.76 ppm. In addition, the ^13^C-NMR of **17** and **18** showed the carbonyl carbons at *δ* 161.42 and 161.35 ppm, respectively.

### 2.6. X-ray Diffraction Analysis

**Figure 2:**
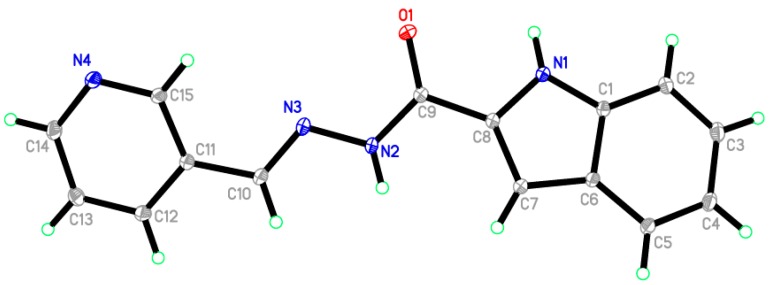
ORTEP diagram of the titled compound **11** drawn at 50% ellipsoids for non-hydrogen atoms.

**Figure 3:**
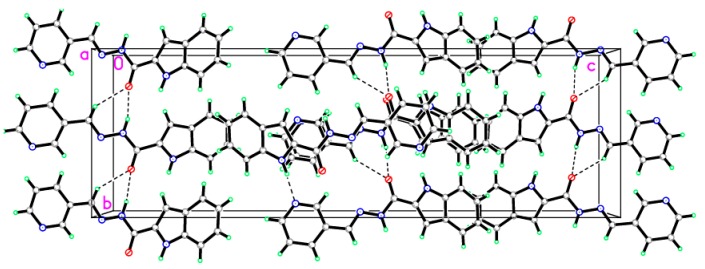
A view of the crystal packing down the *b* axis for the title compound. Dotted lines indicates the intermolecular interaction.

The structure of **13** was confirmed by X-ray crystal structural analysis. The crystallographic data, conditions retained for the intensity data collection and some features of the structure refinements are listed in Table 2. Selected interatomic distances and bond angles are given in Table 3. The unit cell of the titled compound contains one molecule. All of the bond lengths and bond angles in the phenyl rings are in the normal range. The indole ring (C1—C8/N1) forms a dihedral angle of 28.05° with the pyridine-3-yl ring (C11-C14/N4/C15). The title compound exists in *trans* configuration with respect to the C10=N3 bond [1.2805 (15) Å] as shown in Figure 2. In the crystal structure (Figure 3), molecules are linked via three intermolecular N1—H1N1···N4, N2—H1N2···O1 and C10—H10A···O1 hydrogen bonds in *b* axis (Table 4).

## 3. Experimental Section

### 3.1. General Details

Melting points were measured with a Stuart melting-point apparatus (SMP10, Bibby Scientific Ltd., Staffordshire, UK) in open capillaries and are uncorrected. Flash chromatography was done on silica gel 60 (230–400 mesh ASTM). TLC was performed on silica gel 60 F_254_ (Merck Millipore, Darmstadt, Germany) and spots were detected by absorption of UV light. ^1^H-NMR spectra were recorded on Advanced NMR spectrometers (Bruker Biospin, Fallanden, Switzerland) at 300–600 MHz whereas ^13^C-NMR spectra were recorded on the same instruments at 75–150 MHz, with TMS as internal standard. Mass spectra were obtained using MAT312 (ThermoFinnigan GmbH, Tokyo, Japan) and a JMS.600H (Jeol, Tokyo, Japan) instruments for EIMS; HRMS spectra were recorded on a Thermo Finnigan MAT 95XP and Jeol JMS HX110 and ESI on an Ion Trap 6320 mass detector (Agilent Technologies, Wilmington, DE, USA). IR spectra were recorded using KBr discs on a Bruker FT-IR IFS 48 spectrophotometer (Bruker Optics, Ettlingen, Germany).

### 3.2. General Procedure for the Alkylation of Ethyl Indol-2-carboxylate (***1***)

A solution of ethyl indol-2-carboxylate (**1**, 1.0 mmol) and aq. KOH (3.0 mmol) in acetone (10 mL) was stirred at 20 °C for half hour, then the appropriate alkylating agent (1.1 mmol) was added and stirring was continued for 2 h to give **2** and **3** and for eight hours to give **4**. The solvent was removed, water was added and organic layer was extracted using ethyl acetate. The products were purified using column chromatography (ethyl acetate/hexane 1:9).

### 3.3. Hydolysis of the Ester and Formation of Acids ***5***–***7***

*Method a:* the above procedure was followed until the alkylation was complete, then KOH (6.0 mmol in 1.0 mL·H_2_O) was added and the reaction mixture refluxed one hour, the solvent removed, cold water added, and acidified. The ppt was collected and purified by crystallization from ethanol in the case of **5** and **6** and from hexane in the case of **7**.

*Method b:* a solution of the appropriate ester **2**–**4** and KOH (6.0 mmol in 1.0 mL·H_2_O) and acetone (10 mL) was refluxed for one hour, then the above purification process was followed.

*Method c:* A mixture of ethyl indol-2-carboxylate (**1**, 1.0 mmol) and NaOEt (3.0 mmol) in ethanol (10 mL) was stirred for half an hour then, alkylating agent was added and the mixture heated under reflux for two hours and the above purification process followed.

*Ethyl 1-allyl-1H-indole-2-carboxylate* (**2**): Colorless oil, R_f_ 0.65 (ethyl acetate/*n*-hexane 1:9); ^1^H-NMR (DMSO-*d*_6_, 600 MHz) δ 1.33 (t, 3H, *J* 6.3 Hz, CH_3_), 4.32 (q, 2H, OCH_2_CH_3_), 4.81 (d, 1H, *J*_trans_ 16.8 Hz, -NCH_2_-CH=CHH), 5.06 (d, 1H, *J*_cis_ 10.2 Hz, -NCH_2_-CH=CHH), 5.23 (s, 2H, -NCH_2_-CH=CH_2_), 5.97–6.07 (m, 1H, -NCH_2_-CH=CHH), 7.15 (dd, 1H, *J*_4,5_ 7.8, *J*_5,6_ 7.2 Hz, H-5_Indol_), 7.32–7.34 (m, 2H, H-3_Indol_, H-6_Indol_), 7.56 (d, 1H, *J*_6,7_ 8.4 Hz, H-7_Indol_), 7.71 (d, 1H, *J*_4,5_ 7.8 Hz, H-4_Indol_); ^13^C-NMR (DMSO-*d*_6_, 150 MHz) δ 14.7 (CH_3_), 46.7 (-NCH_2_-CH=CH_2_), 60.9 (OCH_2_CH_3_), 110.7 (C-3_Indol_), 111.6 (C-7_Indol_), 116.2 (-NCH_2_-CH=CH_2_), 121.1 (C-5_Indol_), 122.9 (C-4_Indol_), 125.5 (C-6_Indol_), 125.9 (C-2_Indol_), 127.6 (C-3a_Indol_), 135.0 (-NCH_2_-CH=CH_2_), 139.2 (C-7a_Indol_), 161.6 (C=O); LRMS-ESI^+^ *m*/*z* (int. %): 58.7 (6), 79.3 (55), 101.1 (11), 142.1 (100), 182.0 (10), 216.9 (31), 230 (18 for M + H).

*Ethyl 1-benzyl-1H-indole-2-carboxylate* (**3**): White ppt, m.p. 52–53 °C (lit. [26] 55–56 °C), R*_f_* 0.61 (ethyl acetate/*n*-hexane 1:9); ^1^H-NMR (DMSO-*d*_6_, 600 MHz) δ 1.28 (t, 3H, *J* 7.2 Hz, CH_3_), 4.27 (q, 2H, OC*H*_2_CH_3_), 5.87 (s, 2H, -NC*H*_2_-Ph), 7.05 (d, 2H, *J* 7.8 Hz, 2 H_Ph_), 7.14–7.33 (m, 5H, H-5_Indol_, H-6_Indol_, 3 H_Ph_), 7.40 (s, 1H, H-3_Indol_), 7.57 (d, 1H, *J*_6,7_ 8.4 Hz, H-7_Indol_), 7.74 (d, 1H, *J*_4,5_ 7.8 Hz, H-4_Indol_); ^13^C-NMR (DMSO-*d*_6_, 150 MHz) δ 14.5 (OCH_2_*C*H_3_), 47.6 (N*C*H_2_-Ph), 60.9 (O*C*H_2_CH_3_), 111.1, 111.8 (C-3_Indol_, C-7_Indol_), 121.3 (C-5_Indol_), 123.0 (C-4_Indol_), 125.7 (C-6_Indol_), 126.1 (C-2_Indol_), 126.7 (2 CH_Ph_), 127.5 (CH_Ph_) 127.7 (C-3a_Indol_), 128.9 (2 CH_Ph_), 138.9, 139.6 (C_Ph_, C-7a_Indol_), 161.7 (C=O).

*Ethyl 1-pentyl-1H-indole-2-carboxylate* (**4**): Colorless oil, R*_f_* 0.75 (ethyl acetate/*n*-hexane 1:9); ^1^H-NMR (DMSO-*d*_6_, 600 MHz) δ 0.83 (t, 3H, *J* 6.3 Hz, CH_3_), 1.23–1.29 (m, 4H, 2 CH_2_), 1.34 (t, 3H, *J* 6.6 Hz, OCH_2_C*H*_3_), 1.68–1.70 (m, 2H, CH_2_), 4.33 (q, 2H, OC*H*_2_CH_3_), 4.55 (t, 2H, *J* 6.6 Hz, -NC*H*_2_-), 7.13 (dd, 1H, *J*_4,5_ 7.8, *J*_5,6_ 7.2 Hz, H-5_Indol_), 7.28 (s, 1H, H-3_Indol_), 7.34 (dd, 1H, *J*_5,6_ 7.2, *J*_6,7_ 8.4 Hz, H-6_Indol_), 7.59 (d, 1H, *J*_6,7_ 8.4 Hz, H-7_Indol_), 7.68 (d, 1H, *J*_4,5_ 7.8 Hz, H-4_Indol_); ^13^C-NMR (DMSO-*d*_6_, 150 MHz) δ 14.3, 14.6 (2 CH_3_), 22.3, 28.8, 30.4 (3 CH_2_), 44.4 (N*C*H_2_-), 60.8 (O*C*H_2_CH_3_), 110.1, 111.4 (C-3_Indol_, C-7_Indol_), 120.9 (C-5_Indol_), 122.8 (C-4_Indol_), 125.4 (C-6_Indol_), 125.8 (C-2_Indol_), 127.5 (C-3a_Indol_), 139.2 (C-7a_Indol_), 161.7 (C=O).

*1-Allyl-1H-indole-2-carboxylic acid* (**5**): White solid, m.p. 178–179 °C, R*_f_* 0.51 (ethyl acetate/*n*-hexane 3:7); ^1^H-NMR (DMSO-*d*_6_, 600 MHz) δ 4.81 (d, 1H, *J*_trans_ 17.4 Hz, -NCH_2_-CH=C*H*H), 5.05 (d, 1H, *J*_cis_ 10.2 Hz, -NCH_2_-CH=CH*H*), 5.24 (d, 2H, *J* 1.2 Hz, -NC*H*_2_-CH=CH_2_), 5.96–6.00 (m, 1H, -NCH_2_-C*H*=CHH), 7.13 (dd, 1H, *J*_4,5_ 7.8, *J*_5,6_ 7.2 Hz, H-5_Indol_), 7.27 (s, 1H, H-3_Indol_), 7.32 (dd, 1H, *J*_5,6_ 7.2, *J*_6,7_ 8.4 Hz, H-6_Indol_), 7.53 (d, 1H, *J*_6,7_ 8.4 Hz, H-7_Indol_), 7.69 (d, 1H, *J*_4,5_ 7.8 Hz, H-4_Indol_), 12.92 (br.s, 1H, -COOH); ^13^C-NMR (DMSO-*d*_6_, 150 MHz) δ 46.5 (-N*C*H_2_-CH=CH_2_), 110.5, 111.5 (C-3_Indol_, C-7_Indol_), 116.1 (-NCH_2_-CH=*C*H_2_), 120.9 (C-5_Indol_), 122.9 (C-4_Indol_), 125.2 (C-6_Indol_), 126.0 (C-2_Indol_), 128.4 (C-3a_Indol_), 135.2 (-NCH_2_-*C*H=CH_2_), 139.2 (C-7a_Indol_), 163.2 (C=O); LRMS-ESI^−^ *m*/*z* (int. %): 116.0 (20), 155.9 (50), 199.9 (100 for M − H).

*1-Benzyl-1H-indole-2-carboxylic acid* (**6**): White solid, m.p. 190–191 °C (lit. [26] 194–196 °C), R*_f_* 0.50 (ethyl acetate/*n*-hexane 3:7); ^1^H-NMR (DMSO-*d*_6_, 600 MHz) δ 5.89 (s, 2H, -NC*H*_2_-Ph), 7.04 (d, 2H, *J* 6.6 Hz, 2 H_Ph_), 7.12–7.29 (m, 5H, H-5_Indol_, H-6_Indol_, 3 H_Ph_), 7.34 (s, 1H, H-3_Indol_), 7.54 (d, 1H, *J*_6,7_ 8.4 Hz, H-7_Indol_), 7.72 (d, 1H, *J*_4,5_ 7.8 Hz, H-4_Indol_), 12.99 (br.s, 1H, -COOH); ^13^C-NMR (DMSO-*d*_6_, 150 MHz) δ 47.4 (N*C*H_2_-Ph), 110.9, 111.7 (C-3_Indol_, C-7_Indol_), 121.1 (C-5_Indol_), 122.8 (C-4_ndol_), 125.4 (C-6_Indol_), 126.1 (C-2_Indol_), 126.7 (2 CH_Ph_), 127.4 (CH_Ph_) 128.7 (C-3a_Indol_), 128.9 (2 CH_Ph_), 139.1, 139.4 (C_Ph_, C-7a_Indol_), 163.4 (C=O); LRMS-ESI^+^ *m*/*z* (int. %): 142.1 (10), 156.9 (30), 169.9 (11), 178.8 (13), 252.0 (100 for M + H).

*1-Pentyl-1H-indole-2-carboxylic acid* (**7**): White solid, m.p. 91–92 °C, R*_f_* 0.43 (ethyl acetate/*n*-hexane 3:7); ^1^H-NMR (CDCl_3_, 400 MHz) δ 0.93 (t, 3H, *J* 6.9 Hz, CH_3_), 1.28–1.42 (m, 4H, 2 CH_2_), 1.82–1.89 (m, 2H, CH_2_), 4.61 (t, 2H, *J* 7.5 Hz, NCH_2_), 7.19 (dd, 1H, *J*_5,6_ 7.4, *J*_4,5_ 8.0 Hz, H-5_Indol_), 7.40 (dd, 1H, *J*_5,6_ 7.4, *J*_6,7_ 8.1 Hz, H-6_Indol_), 7.45 (d, 1H, *J*_6,7_ 8.1 Hz, H-7_Indol_), 7.51 (s, 1H, H-3_Indol_), 7.74 (d, 1H, *J*_4,5_ 8.0 Hz, H-4_Indol_); IR (KBr): ν_max_/cm^−1^ 2500–3500 (OH _acid_), 1684.1 (C=O _acid_).

### 3.4. Transesterification Procedures

A mixture of ethyl indol-2-carboxylate **1** (1.0 mmol) with or without the alkylating agents and NaOMe (4.0 mmol) in methanol (10 mL) was stirred for one hour. The mixture was acidified and the precipitate was collected, dried and crystallized from ethanol or purified by sublimation.

*Methyl 1H-indole-2-carboxylate* (**8**): Yield: 89% as colorless needle crystals, m.p. 149–150 °C, R*_f_* 0.28 (ethyl acetate/*n*-hexane 1:9); ^1^H-NMR (DMSO-*d*_6_, 600 MHz) δ 3.88 (s, 3H, CH_3_), 7.09 (dd, 1H, *J*_4,5_ 7.8, *J*_5,6_ 7.2 Hz, H-5_Indol_), 7.18 (s, 1H, H-3_Indol_), 7.27 (dd, 1H, *J*_5,6_ 7.2, *J*_6,7_ 8.4 Hz, H-6_Indol_), 7.49 (d, 1 H, *J*_6,7_ 8.4 Hz, H-7_Indol_), 7.66 (d, 1H, *J*_4,5_ 7.8 Hz, H-4_Indol_), 11.91 (s, 1H, NH_Indol_); ^13^C-NMR (DMSO-*d*_6_, 150 MHz) δ 52.2 (CH_3_), 108.3 (C-3_Indol_), 113.1 (C-7_Indol_), 120.7 (C-5_Indol_), 122.5 (C-4_Indol_), 125.1 (C-6_Indol_), 127.2, 127.5 (C-2_Indol_, C-3a_Indol_), 137.9 (C-7a_Indol_), 162.3 (C=O); LRMS-ESI^−^ *m*/*z* (int. %): 89.0 (6), 113.1 (7), 145.9 (5), 158.9 (10), 173.9 (100 for M − H).

### 3.5. Synthesis of ***9*** Following Methods b: Starting with ***1*** or ***8***

*1H-Indole-2-carboxylic acid* (**9**): Yield: 55% as yellow solid, m.p. 203–204 °C, R*_f_* 0.30 (ethyl acetate/*n*-hexane 3:7); ^1^H-NMR (DMSO-*d*_6_, 600 MHz) δ 7.06 (dd, 1H, *J*_4,5_ 7.8, *J*_5,6_ 7.2 Hz, H-5_Indol_), 7.11 (s, 1H, H-3_Indol_), 7.24 (dd, 1H, *J*_5,6_ 7.2, *J*_6,7_ 8.4 Hz, H-6_Indol_), 7.46 (d, 1H, *J*_6,7_ 8.4 Hz, H-7_Indol_), 7.65 (d, 1H, *J*_4,5_ 7.8 Hz, H-4_Indol_), 11.74 (s, 1H, NH_Indol_); ^13^C-NMR (DMSO-*d*_6_, 150 MHz) δ 107.79, 112.96 (C-3_Indol_, C-7_Indol_), 120.4 (C-5_Indol_), 122.4 (C-4_ndol_), 124.7 (C-6_Indol_), 127.3, 128.9 (C-2_Indol_, C-3a_Indol_), 137.7 (C-7a_Indol_), 163.3 (C=O); LRMS-ESI^−^ *m*/*z* (int. %): 115.9 (25), 159.8 (100 for M + H).

### 3.6. Hydrazide Formation

Either ethyl indol-2-carboxylate **1** or methyl indol-2-carboxylate **8** was refluxed with hydrazine hydrate in ethanol (4 h), the formed ppt was collected and crystalized from 95% ethanol.

*1H-Indole-2-carbohydrazide* (**10**): Yield: 90% as colorless crystals, m.p. 247–248 °C (lit. [17,18]), R*_f_* 0.43 (9:1 CH_2_Cl_2_/MeOH); ^1^H-NMR (DMSO-*d*_6_, 600 MHz): δ 4.52 (s, 2H, NH_2_), 7.03 (dd, 1H, *J*_5,6_ 7.2, *J*_4,5_ 7.8 Hz, H-5_Indol_), 7.10 (s, 1H, H-3_Indol_), 7.18 (dd, 1H, *J*_5,6_ 7.2, *J*_6,7_ 7.8 Hz, H-6_Indol_), 7.45 (d, 1H, *J*_6,7_ 7.8 Hz, H-7_Indol_), 7.60 (d, 1H, *J*_4,5_ 7.8 Hz, H-4_Indol_), 9.78 (s, 1H, NH), 11.60 (s, 1H, NH_Indol_); ^13^C-NMR (DMSO-*d*_6_, 150 MHz): δ 102.3 (C-3_Indol_), 112.7 (C-7_Indol_), 120.2 (C-5_Indol_), 121.9 (C-4_Indol_), 123.6 (C-6_Indol_), 127.6, 131.0, 136.8 (C-2_Indol_, C-3a_Indol_, C-7a_Indol_), 161.7 (C=O). LRMS-ESI^−^ *m*/*z* (Int. %): 115.9 (6), 173.9 (100 for M − H).

*N′-β-d-Glucopyranosyl-1H-indole-2-carbohydrazide* (**11**): Yield: 60% as white solid, m.p. 208–210 °C, R*_f_* 0.10 (MeOH/DCM 1.5:8.5); ^1^H-NMR (DMSO-*d*_6_ + D_2_O, 300 MHz) δ 3.02–3.24 (m, 4H, H-5_Glc_, H-6_Glc_, H-3_Glc_, H-4_Glc_), 3.44 (dd, 1H, *J*_5,6_ 6, *J*_6,6′_ 17.7 Hz, H-6′_Glc_), 3.66 (dd, 1H, *J* 9.9 Hz, H-2_Glc_), 3.87 (d, 1H, *J* 8.7 Hz, H-1_Glc_), 7.02 (dd, 1H, *J*_4,5_ 8.1, *J*_5,6_ 7.2 Hz, H-5_Indol_), 7.16–7.23 (m, 2H, H-3_Indol_, H-6_Indol_), 7.42 (d, 1H, *J*_6,7_ 8.1 Hz, H-7_Indol_), 7.59 (d, 1H, *J*_4,5_ 8.1 Hz, H-4_Indol_); ^13^C-NMR (DMSO-*d*_6_ + D_2_O, 150 MHz) δ 61.3, 70.3, 71.2, 76.6, 78.1, 90.9 (6 C_Glc_), δ 103.3 (C-3_Indol_), 112.25 (C-7_Indol_), 119.8 (C-2_Indol_), 121.5 (C-5_Indol_), 123.4 (C-4_ndol_), 126.9, 129.7, 136.5 (C-6_Indol_, C-3a_Indol_, C-7a_Indol_), 161.3 (C=O); HRMS (+ESI) calcd for C_15_H_20_N_3_O_6_ [M^+^]: 338.1352. Found: 338.1348.

### 3.7. Condensation of Hydrazide with Aromatic Aldehydes and Ketones

A mixture of hydrazide **10** (1.0 mmol) and the appropriate aldehyde or ketone (1.1 mmol) in ethanol (5.0 mL) containing acetic acid (0.5 mL) was refluxed until the ppt appeared. The ppt was filtered and crystalized from DMF or DMF/EtOH mixture.

*N'-((1H-Indol-3-yl)methylene)-1H-indole-2-carbohydrazide* (**12**): Yield: 82% as yellowish white needle-like crystals, m.p. 277–278 °C, R*_f_* 0.49 (ethyl acetate/*n*-hexane 6:4); ^1^H-NMR (DMSO-*d*_6_, 600 MHz): δ 7.08–7.23 (m, 5H), 7.30–7.49 (m, 2H), 7.68 (d, 1H, *J* 6.6 Hz), 7.86 (s, 1H), 8.32 (d, 1H, *J* 6.0 Hz), 8.65 (s, 1H), 11.56, 11.61, 11.70 (3s, 1H, 3 NH); ^13^C-NMR (DMSO-*d*_6_, 150 MHz): δ 103.3, 112.2, 112.3, 112.8, 120.3, 120.9, 122.06, 122.4, 123.1, 123.9, 124.8, 127.6, 130.7, 131.2, 137.2, 137.5, 145.0, 157.6 (C=O); LRMS-ESI^+^ *m*/*z* (Int. %): 79.4 (6), 303.1 (100 for M + H).

*N′-(Pyridin-3-ylmethylene)-1H-indole-2-carbohydrazide* (**13**): Yield: 71% as colorless needle crystals, m.p. 250–251 °C, R*_f_* 0.35 (ethyl acetate/*n*-hexane 6:4); ^1^H-NMR (DMSO-*d*_6_, 600 MHz): δ 7.07–7.70 (m, 6H), 8.18 (d, 1H, *J* 4.8 Hz), 8.52 (s, 1H), 8.63 (s, 1H), 8.90 (s, 1H), 11.83, 12.07 (2 s, 2H, NH, NH_Indol_); ^13^C-NMR (DMSO-*d*_6_, 150 MHz): δ 104.3 (C-3_Indol_), 112.9 (C-7_Indol_), 120.5 (C-5_Indol_), 122.3 (C-4_Indol_), 124.5 (C-6_Indol_, CH_Pyridin_), 127.4, 130.5, 130.8, 133.9, 137.4, 144.8, 151.1, 158.2 (C-2_Indol_, C-3a_Indol_, C-7a_Indol_, 3 CH_Pyridin_, 2 C_Pyridin_), 160.8 (C=O). LRMS-ESI^−^ *m*/*z* (Int. %): 79.3 (8), 265.0 (100 for M + H).

*N'-(1-(2-Aminophenyl)ethylidene)-1H-indole-2-carbohydrazide* (**14**): Yield: 60% as colorless scale crystals, m.p. 207–209 °C, R*_f_* 0.82 (ethyl acetate/*n*-hexane 6:4); ^1^H-NMR (DMSO-*d*_6_, 600 MHz): δ 2.43 (s, 3H, CH_3_), 6.56 (t, 1H, *J* 7.2 Hz), 6.77 (d, 1H, *J* 7.8 Hz), 7.07–7.09 (m, 2H), 7.23–7.26 (m, 2H), 7.41 (s, 1H), 7.48 (d, 1H, *J* 7.8 Hz), 7.50 (d, 1H, *J* 8.4 Hz), 7.69 (d, 1H, *J* 7.8 Hz), 10.84 (s, H, NH), 11.79 (s, H, NH_Indol_); ^13^C-NMR (DMSO-*d*_6_, 150 MHz): δ 15.5 (CH_3_), 104.9, 112.8, 115.0, 116.6, 118.1, 120.4, 122.2, 124.3, 127.5, 129.6, 130.1, 130.6, 148.5, 157.3, 158.7 (2 C=O).

*N′,N′′-(1,4-Phenylenebis(methan-1-yl-1-ylidene))bis(1H-indole-2-carbohydrazide)* (**15**): Yield: 90% as yellowish white solid, m.p. 332–333 °C, R*_f_* 0.77 (ethyl acetate); ^1^H-NMR (DMSO-*d*_6_, 600 MHz): δ 7.09 (dd, 2H, *J*_5,6_ 7.2, *J*_4,5_ 7.8 Hz, 2 H-5_Indol_), 7.25 (dd, 2H, *J*_5,6_ 7.2, *J*_6,7_ 6.6 Hz, 2 H-6_Indol_), 7.25 (s, 2H, 2 H-3_Indol_), 7.50 (d, 2H, *J*_6,7_ 7.8 Hz, 2 H-7_Indol_), 7.70 (br, 2H, 2 H-4_Indol_), 7.87 (s, 4H, 4 H_Ph_), 8.51 (s, 2H, -N=CH), 11.86 (s, 2H, 2 NH_Indol_), 12.02 (s, 2H, 2 NH); ^13^C-NMR (DMSO-*d*_6_, 150 MHz): δ 104.30 (2 C-3_Indol_), 112.9 (2 C-7_Indol_), 120.5 (2 C-5_Indol_), 122.3 (2 C-4_Indol_), 124.4 (2 C-6_Indol_), 127.5, 128.0, 130.5 (2 C-2_Indol_, 2 C-3a_Indol_, 4 CH_Ph_), 136.2, 137.4 (2 C_Ph_, 2 C-7a_Indol_), 146.9, 158.2 (2 CH=N, 2 C=O). LRMS-ESI^+^ *m*/*z* (Int. %): 79.3 (100), 101.0 (20), 142.1 (62), 237.0 (66), 280.0 (30), 341.9 (12), 448.8 (10 for M + H).

### 3.8. Synthesis of N′-(4-Aryl-1,3-thiazol-2-yl)-1H-indole-2-carbohydrazides ***17***, ***18***

A mixture of 1-[(1*H*-Indol-2-yl)-carbonyl]-thiosemicarbazide (**16** [29], 1.0 mmol) and the respective phenacyl bromide (1.1 mmol) in ethanol (10 mL) was stirred at room temperature for 25 min. A precipitate was formed, filtered, and then recrystallized from ethanol.

*N′-(4-Phenyl-1,3-thiazol-2-yl)-1H-indole-2-carbohydrazide* (**17**): Yield: 68% as pink shiny crystals, m.p. 245–247 °C, R*_f_* 0.68 (ethyl acetate/*n*-hexane 6:4); ^1^H-NMR (DMSO-*d*_6_, 400 MHz) δ 7.07 (dd, 1H, *J*_4,5_ 8.0, *J*_5,6_ 7.6 Hz, H-5_Indol_), 7.19–7.29 (m, 4H, H-3_Indol_, H-6_Indol_, H-4_Thiazol_, CH_Ph_), 7.38 (dd, 2H, *J* 7.2, *J* 8.0 Hz, 2H_Ph_), 7.45 (d, 1H, *J*_6,7_ 8.0 Hz, H-7_Indol_), 7.66 (d, 1H, *J*_4,5_ 8.0 Hz, H-4_Indol_), 7.83 (d, 2H, *J* 7.6 Hz, 2H_Ph_), 9.69 (br. s, 1H, NH), 10.87 (br. s, 1H, NH), 11.76 (br. s, 1H, NH_Indol_); ^13^C-NMR (DMSO-*d*_6_, 100 MHz) δ 103.16, 103.69 (C-3_Indol_, CH_Thiazol_), 112.43 (C-7_Indol_), 120.06 (C-5_Indol_), 121.83 (C-4_Indol_), 123.93 (C-6_Indol_), 125.60 (2 CH_Ph_), 126.98 (C-3a_Indol_), 127.53 (CH_Ph_), 128.60 (2 CH_Ph_), 129.19 (C-2_Indol_), 134.69 (C_Ph_), 136.8 (C-7a_Indol_), 150.71 (C-4_Thiazol_), 161.42 (C=O), 172.71 (C-2_Thiazol_)); HRMS (EI) calcd for C_18_H_14_N_4_OS [M^+.^]: 334.0888. Found: 334.0890.

*N′-(4-(3-Bromophenyl)-1,3-thiazol-2-yl)-1H-indole-2-carbohydrazide* (**18**): Yield: 60% as pink shiny crystals, m.p. 250–251 °C, R*_f_* 0.73 (ethyl acetate/n-hexane 6:4); ^1^H-NMR (DMSO-*d*_6_, 300 MHz) δ 7.07 (dd, 1H, *J*_4,5_ 7.8, *J*_5,6_ 7.5 Hz, H-5_Indol_), 7.19–7.27 (m, 2H, H-3_Indol_, H-6_Indol_), 7.35 (dd, 1H, *J* 7.8, *J* 8.1 Hz, CH_Ph_), 7.42–7.48 (m, 3H, H-7_Indol_, H-4_Thiazol_, CH_Ph_), 7.66 (d, 1H, *J*_4,5_ 7.8 Hz, H-4_Indol_), 7.84 (d, 1H, *J* 7.5 Hz, 2H_Ph_), 8.02 (s, 1H, CH_Ph_), 9.75 (br. s, 1H, NH), 10.89 (br. s, 1H, NH), 11.77 (br. s, 1H, NH_Indol_); ^13^C-NMR (DMSO-*d*_6_, 100 MHz) δ 103.67, 104.71 (C-3_Indol_, CH_Thiazol_), 112.37 (C-7_Indol_), 120.00 (C-5_Indol_), 121.78 (C-4_Indol_), 122.02 (C_Ph_), 123.90 (C-6_Indol_), 124.42 (CH_Ph_), 126.90 (C-3a_Indol_), 128.09 (CH_Ph_), 129.05 (C-2_Indol_), 130.30, 130.76 (2 CH_Ph_), 134.75, 136.84 (C-7a_Indol_, C_Ph_), 148.87 (C-4_Thiazol_), 161.35 (C=O), 172.81 (C-2_Thiazol_); HRMS (EI) calcd for C_18_H_13_N_4_OSBr [M^+^]: 411.9993. Found: 411.9954.

### 3.9. X-ray Crystallography

Compound **13** was obtained as single crystals by slow evaporation of ethanol. Data were collected on a Bruker APEX-II D8 Venture area diffractometer (Bruker AXS GmbH, Karlsruhe, Germany), equipped with graphite monochromatic Mo Kα radiation at 100(2) K. Cell refinement and data reduction were carried out by Bruker SAINT. SHELXS-97 [33,34,35,36] was used to solve structure. The final refinement was carried out by full-matrix least-squares techniques with anisotropic thermal data for nonhydrogen atoms on *F*^2^. CCDC 1438576 contains the supplementary crystallographic data for this paper. These data can be obtained free of charge via http://www.ccdc.cam.ac.uk/conts/retrieving.html (or from the CCDC, 12 Union Road, Cambridge CB2 1EZ, UK; Fax: +44 1223 336033; E-mail: deposit@ccdc.cam.ac.uk).

## 4. Conclusions

In summary, using NaOMe did not catalyze the alkylation of the ethyl indole-2-carboxylate (NH), and instead led to transesterification. The alkylation succeeded by the use of aq. KOH in acetone and is time dependent. Hydrazinolysis of ethyl or methyl indol-2-carboxylate afforded indol-2-carbohydrazide, which reacted with some aromatic aldehydes and ketones to form hydrazones. Indol-2-thiosemicarbazide was used for the synthesis of thiazoles. Further biological evaluations of the synthesized compounds **2**–**18** are underway.

## Figures and Tables

**Table 1:** Conditions of indole nitrogen alkylation and ester hydrolysis.

Entry	Reactant	R-Br	Base	T (°C)	Time (h)	Product	Yield (%)
1	1	Allyl-Br	KOH (3.0 mmol/0.1 mL·H_2_O)	20	2.0	**2**	85
2	1	Benzyl-Br	KOH (3.0 mmol/0.1 mL·H_2_O)	20	2.0	**3**	94
3	1	Amyl-Br	KOH (3.0 mmol/0.1 mL·H_2_O)	20	8.0	**4/9**	60/30
4	2	-	KOH (6.0 mmol/1.0 mL·H_2_O)	60	1.0	**5**	95
5	3	-	KOH (6.0 mmol/1.0 mL·H_2_O)	60	1.0	**6**	97
6	4	-	KOH (6.0 mmol/1.0 mL·H_2_O)	60	1.0	**7**	90
7	1	Allyl-Br	NaOEt (6.0 mmol)/EtOH	60	2	**5**	35
8	1	Benzyl-Br	NaOEt (6.0 mmol)/EtOH	60	2	**6**	40
9	1	Amyl-Br	NaOEt (6.0 mmol)/EtOH	60	2	**9**	90

**Table 2:** The crystal structure and refinement data of compound **13**.

**Crystal Data**	
Chemical formula	C_15_H_12_N_4_O
M*r*	264.29
Crystal system, space group	Orthorhombic, *Pbca*
Temperature (K)	100
*a, b, c* (Å)	7.8503 (2), 10.1780 (3), 31.9226 (9)
*V* (Å^3^)	2550.63 (12)
*Z*	8
Radiation type	Mo Kα
µ (mm^−1^)	0.09
Crystal size (mm)	0.47 × 0.35 × 0.23
**Data Collection**	
Diffractometer	Bruker APEX-II D8 venture diffractometer
Absorption correction	Multi-scan, SADABS Bruker 2014
T_min_, T_max_	0.844, 0.881
No. of measured, independent and observed [I > 2σ(I)] reflections	17978, 2927, 2645
Rint	0.032
**Refinement**	
R[*F*^2^ > 2σ(*F*^2^)], wR(*F*^2^), S	0.037, 0.104, 1.04
No. of reflections	2927
No. of parameters	190
No. of restraints	0
H-atom treatment	H atoms treated by a mixture of independent and constrained refinement
Δρ_max_, Δρ_min_ (e·Å^−3^)	0.36, −0.22

**Table 3:** Selected bond lengths and bond angles in compound **13**.

Atoms	Å, °	Atoms	Å, °
O1—C9	1.2301 (14)	N2—C9	1.3594 (15)
N1—C1	1.3700 (14)	N3—C10	1.2805 (15)
N1—C8	1.3773 (15)	N4—C14	1.3430 (16)
N2—N3	1.3771 (14)	N4—C15	1.3376 (14)
C1—N1—C8	108.34 (9)	N1—C8—C9	117.81 (10)
N3—N2—C9	119.20 (9)	O1—C9—C8	121.67 (10)
N2—N3—C10	115.30 (10)	N2—C9—C8	114.68 (10)
C14—N4—C15	117.59 (10)	O1—C9—N2	123.62 (10)
N1—C1—C6	108.28 (9)	N3—C10—C11	119.51 (10)
N1—C1—C2	129.58 (11)	N4—C14—C13	122.99 (12)
N1—C8—C7	110.20 (9)	N4—C15—C11	123.74 (10)

**Table 4:** Hydrogen-bond geometry (Å, °) in compound **13**.

*D*—H···*A*	*D*—H	H···*A*	*D*···*A*	*D*—H···*A*
N1—H1N1···N4^i^	0.915(17)	1.975(17)	2.8856(14)	173.4(14)
N2—H1N2···O1^ii^	0.915(17)	2.135(17)	2.9909(13)	155.4(14)
C10—H10A···O1^ii^	0.9300	2.4300	3.2266(14)	144.00
Symmetry codes: (i) *x* − 1/2, −*y* + 3/2, −*z* + 1; (ii) –*x* + 3/2, *y* + 1/2, *z*.				

## References

[1] Sundberg R.J. (1996). Indoles.

[2] Robert M.F., Wink M. (1998). Alkaloids: Biochemistry, Ecology, and Medicinal Applications.

[3] Von Nussbaum F. (2003). Stephacidin B—A new stage of complexity within prenylated indole alkaloids from fungi. Angew. Chem. Int. Ed..

[4] Pindur U., Lemster T. (2001). Advances in marine natural products of the indole and annelated indole series: Chemical and biological aspects. Curr. Med. Chem..

[5] Hesse M. (2002). Alkaloids. Nature’s Curse or Blessing.

[6] Sharma V., Kumar P., Pathak D. (2010). Biological importance of the indole nucleus in recent years: A comprehensive review. J. Heterocycl. Chem..

[7] Premanathan M., Radhakrishnan S., Kulangiappar K., Singaravelu G., Thirumalaiarasu V., Sivakumar T., Kathiresan K. (2012). Antioxidant & anticancer activities of isatin (1*H*-indole-2,3-dione), isolated from the flowers of *Couroupita guianensis* Aubl. Indian J. Med. Res..

[8] Islam M.R., Mohsin M. (2007). Synthesis of isatin, 5-chloroisatin and their Δ^2^–1, 3, 4 oxadiazoline derivatives for comparative cytotoxicity study on brine shrimp. Bangladesh J. Pharmacol..

[9] Matesic L., Locke J.M., Bremner J.B., Pyne S.G., Skropeta D., Ranson M., Vine K.L. (2008). *N*-phenethyl and *N*-naphthylmethyl isatins and analogues as *in vitro* cytotoxic agents. Bioorg. Med. Chem..

[10] Gao N., Kramer L., Rahmani M., Dent P., Grant S. (2006). The three-substituted indolinone cyclin-dependent kinase 2 inhibitor 3-[1-(3*H*-imidazol-4-yl)-meth-(*Z*)-ylidene]-5-methoxy-1,3-dihydroindol-2-one (SU9516) kills human leukemia cells via down-regulation of Mcl-1 through a transcriptional mechanism. Mol. Pharmacol..

[11] Wang F., Fang Y., Zhu T., Zhang M., Lin A., Gu Q., Zhu W. (2008). Seven new prenylated indole diketopiperazine alkaloids from holothurian-derived fungus Aspergillus fumigatus. Tetrahedron.

[12] Lane M.E., Yu B., Rice A., Lipson K.E., Liang C., Sun L., Wadler S. (2001). A novel cdk2-selective inhibitor, SU9516, induces apoptosis in colon carcinoma cells. Cancer Res..

[13] Patyna S., Laird A.D., Mendel D.B., O'Farrell A.M., Liang C., Guan H., Grazzini M. (2006). SU14813: a novel multiple receptor tyrosine kinase inhibitor with potent antiangiogenic and antitumor activity. Mol. Cancer Ther..

[14] Li H.H., Zhang X.H., Tan J.Z., Chen L.L., Liu H., Luo X.M., Jiang H.L. (2007). Design, synthesis, antitumor evaluations and molecular modeling studies of novel 3,5-substituted indolin-2-one derivatives. Acta Pharmacol. Sin..

[15] Juranić Z., Anastasova F., Juranić I., Stanojković T., Radulović S., Vuletić N. (1999). Antiproliferative action of isatine-beta-thiocarbohydrazone and N-ethylisatine-beta-thiocarbohydrazone on human PBMC and on two neoplastic cell lines. Exp. Clin. Cancer Res..

[16] Terry A.V., Buccafusco J.J. (2003). The cholinergic hypothesis of age and Alzheimer’s disease-related cognitive deficits: Recent challenges and their implications for novel drug development. J. Pharmacol. Exp. Ther..

[17] Brandes J.L., Kudrow D., Cady R., Tiseo P.J., Sun W., Sikes C.R. (2005). Eletriptan in the early treatment of acute migraine: Influence of pain intensity and time of dosing. Cephalalgia.

[18] Bader T., Fazili J., Madhoun M., Aston C., Hughes D., Rizvi S., Seres K., Hasan M. (2008). Fluvastatin inhibits hepatitis C replication in humans. Am. J. Gastroenterol..

[19] Tramer M.R., Reynolds D.J.M., Moore R.A., McQuay H.J. (1997). Efficacy, Dose-Response, and Safety of Ondansetron in Prevention of Postoperative Nausea and Vomiting A Quantitative Systematic Review of Randomized Placebo-controlled Trials. J. Am. Soc. Anesthesiol..

[20] Mancini I., Guella G., Dbitus C., Waikedre J., Pietra F. (1996). From inactive nortopsentin D, a novel bis (indole) alkaloid isolated from the axinellid sponge Dragmacidon sp. from deep waters south of new caledonia, to a strongly cytotoxic derivative. Helv. Chim. Acta.

[21] Miyake F.Y., Yakushijin K., Horne D.A. (2000). A concise synthesis of topsentin A and nortopsentins B and D. Org. Lett..

[22] Sakemi S., Sun H.H. (1991). Nortopsentins A, B, and C. Cytotoxic and antifungal imidazolediylbis [indoles] from the sponge Spongosorites ruetzleri. J. Org. Chem..

[23] Kawasaki I., Yamashita M., OHTA S. (1996). Total Synthesis of Nortopsentins AD, Marine Alkaloids. Chem. Pharm. Bull..

[24] El-Wareth A., Sarhan A.O. (2001). On the synthesis and reactions of indole-2-carboxylic acid hydrazide. Monatsh. Chem..

[25] Boraei A.T.A. (2016). A new direct synthetic access to 4-amino-2-*N*-(glycosyl/propyl)-1,2,4-triazole-3-thiones via hydrazinolysis of 3-*N*-((acylated glycosyl)/allyl)-1,3,4-oxadiazole-2-thiones. Arkivoc.

[26] El Ashry E.S.H., El Tamany E.S.H., El Fattah M.E.D.A., Aly M.R., Boraei A.T. (2009). Synthesis of New Functionalized 2-Alkylsulfanyl-5-(1*H*-indol-2-yl)-1,3,4-Oxadiazole and a Facile Thio-Aza-Claisen Rearrangement of the S-Allyl Analog. Lett. Org. Chem..

[27] El Ashry E.S.H., Fattah M.E.D.A., Aly M.R., Boraei A.T., Duerkop A. (2003). A new synthetic access to 2-*N*-(glycosyl) thiosemicarbazides from 3-*N*-(glycosyl) oxadiazolinethiones and the regioselectivity of the glycosylation of their oxadiazolinethione precursors. Beilstein J. Org. Chem..

[28] El Ashry E.S.H., El Tamany E.S.H., Abd El Fattah M.E.D., Aly M.R., Boraei A.T., Mesaik M.A., Soomro S. (2013). Immunomodulatory properties of *S*-and *N*-alkylated 5-(1*H*-indol-2-yl)-1,3,4-oxadiazole-2(3*H*)-thione. J. Enzyme Inhib. Med. Chem..

[29] El Ashry E.S.H., El Fattah M.E.D.A., Boraei A.T., El-Nabi H.M.A. (2013). Regioselective synthesis, characterization and antimicrobial evaluation of *S*-glycosides and *S*,*N*-diglycosides of 1,2-dihydro-5-(1*H*-indol-2-yl)-1,2,4-triazole-3-thione. Eur. J. Med. Chem..

[30] Barakat A., Islam M.S., Al Majid A.M.A., Al-Othman Z.A. (2013). Highly enantioselective Friedel–Crafts alkylation of indoles with α, β-unsaturated ketones with simple Cu(II)–oxazoline–imidazoline catalysts. Tetrahedron.

[31] Islam M.I., Al-Majid A.M., Al-Othman Z.A., Barakat A. (2014). Highly enantioselective Friedel-Crafts alkylation of indole with electron deficient trans-*β*-nitroalkenes under simple Zn(II)-oxazoline- imidazoline catalysts. Tetrahedron Asymmetry.

[32] Al-Majid A.M., Islam M.I., Barakat A., Al-Agamy M.H.M., Naushad Mu. (2014). Facile and promising method for Michael addition of indole and pyrrole to electron deficient trans-β-nitroolefins catalyzed by Feist’s acid: Preliminary study of anti-microbial activity. Sci. World J..

[33] Ottoni O., Cruz R., Alves R. (1998). Alves. Efficient and simple methods for the introduction of the sulfonyl, acyl and alky protecting groups on the nitrogen of the indole and its derivatives. Tetrahedron.

[34] Sechi M., Derudas M., Dallocchio R., Dessì A., Bacchi A., Sannia L., Carta F., Palomba M., Ragab O., Chan C. (2004). Design and synthesis of novel indole beta-diketo acid derivatives as HIV-1 integrase inhibitors. J. Med. Chem..

[35] Sheldrick G.M. (2008). A short history of SHELX. Acta Cryst..

[36] Spek A.L. (2009). Structure validation in chemical crystallography. Acta Cryst..

